# Prevalence and predictors of cervicitis in female sex workers in Peru: an observational study

**DOI:** 10.1186/1471-2334-13-195

**Published:** 2013-04-30

**Authors:** Simon Pollett, Martha Calderon, Kristen Heitzinger, Vicky Solari, Silvia M Montano, Joseph Zunt

**Affiliations:** 1 Bacteriology Department, U.S. Naval Medical Research Unit No. 6, Callao, Peru; 2 Sydney Emerging Infections and Biosecurity Institute, University of Sydney, Sydney, Australia; 3 Universidad Nacional Mayor de San Marcos, Lima, Peru; 4 DIRESA Callao, Centro de Salud ‘Alberto Barton’, Lima, Peru; 5 Department of Epidemiology, University of Washington, Seattle, WA, USA; 6 Department of Global Health and Neurology, University of Washington, Seattle, WA, USA

**Keywords:** Cervicitis, FSW, Peru, Bacterial vaginosis, Sexually transmitted infection

## Abstract

**Background:**

Cervicitis is a syndrome of cervical inflammation and a common condition in female sex workers (FSW), a subpopulation vulnerable to sexually transmitted infections. Local data is essential for guiding syndromic management of cervicitis in FSW working in Peru. We sought to describe the prevalence and etiologies of cervicitis in this population. We also aimed to identify sociodemographic, behavioral and biological factors associated with cervicitis, including bacterial vaginosis (BV), a condition with a possible role in cervicitis.

**Methods:**

FSW 18 years of age or older presenting to a free public sexual health clinic in Callao-Lima, Peru were eligible for inclusion upon consent. 467 participants completed a face-to-face questionnaire and underwent genital examination. Vaginal, endocervical and blood samples were collected and tested for *C. trachomatis* (CT), *N. gonorrhea* (GC), *T. vaginalis* (TV), BV, HIV and Human T-Cell Lymphotropic Virus −1. Logistic regression was used to determine whether sociodemographic, behavioral, or other sexual health related characteristics were associated with the diagnosis of cervicitis.

**Results:**

Cervicitis was detected in 99 (24.9%) of 397 FSW. The presence of cervicitis was unable to be determined in 70 participants. In women with cervicitis, CT was present in 4.6% (4/87), TV in 4.0% (4/99), GC in 0% (0/87) and no pathogen was detected on cervical microbiology in 91.9% (91/99). BV was detected on vaginal microbiology in 36.9% (31/84) of cervicitis cases. BV was more common in women with cervicitis, however this association did not reach statistical significance (aOR = 1.47 [0.87, 2.48], p = 0.15). Other STI were not associated with cervicitis. Regular clinic attendance (aOR = 0.54 [0.34, 0.87], p = 0.01) and Ecuadorian nationality (aOR = 0.31 [0.13, 0.76], p = 0.01) were associated with reduced risk of cervicitis.

**Conclusions:**

Cervicitis was common in FSW working Peru and was predominantly nongonococcal and non-chlamydial in etiology. Further study is warranted to clarify the role of BV and other emerging cervicitis pathogens in this population. The current Peruvian program of free health checks for FSW may be effective for reducing rates of cervicitis. The protective effect of Ecuadorian nationality prompts further study.

## Background

Cervicitis is a syndrome of cervical inflammation and a common manifestation of sexually transmitted infections (STI) such as *Chlamydia trachomatis* (CT) and *Neisseria gonorrhoea* (GC) [1,2]. Vaginal discharge or intermenstrual bleeding are frequent symptoms of cervicitis, although it may be asymptomatic [1,3]. Cervicitis can progress to pelvic inflammatory disease (PID) with severe reproductive sequelae, even in asymptomatic cases [3]. Cervicitis may also affect sexual health at a population level, as it increases shedding of other STI such as Human Immunodeficiency Virus (HIV) and Human T-cell Lymphotropic Virus (HTLV) [4,5].

While cervicitis is considered to arise as a consequence of STI, understanding of its etiology and pathogenesis is still evolving. Even with advanced microbiological assays, no causative pathogen is identified in the majority of cervicitis cases. When a pathogen is detected, CT and GC are the usual culprits. Less commonly, *Mycoplasma genitalium*, *Trichomonas vaginalis* (TV) and Herpes simplex Virus (HSV) are implicated [1,2]. More recently, bacterial vaginosis (BV) has been linked to cervicitis, in addition to an increased risk of HIV acquisition, adverse pregnancy outcomes and gynaecological surgery complications [3,6]. Although not an STI *per se*, BV is an endogenous vaginitis associated with sexual activity and characterized by an overgrowth of vaginal anaerobic flora and reduction of H_2_0_2_-producing lactobacilli [3].

Cervicitis is a common condition in FSW, with prevalences as high as 20% [7]. In studies of cervicitis in FSW living in Africa, CT, GC, *M. genitalium* and TV were common pathogens [7-9]. Factors such as lower education level, greater numbers of clients and decreased condom usage were associated with cervical infection in FSW working in Madagascar [10]. Although cervicitis has been reported in Peruvian FSW, detailed data regarding the prevalence, etiologies and risk factors for cervicitis in FSW working in Peru or other Latin American countries is lacking [4,11]. As FSW may receive only intermittent healthcare and advanced diagnostics in low resource settings are limited, cervicitis in FSW, when diagnosed, is most often treated syndromically [12]. Syndromic management has drawbacks such as the underdiagnosis of subclinical STI and a potential overuse of antimicrobials [13]. Since FSW are particularly vulnerable to STI, and cervicitis may increase rates of STI transmission to the general population, accurate local epidemiological data is essential for guiding syndromic management of cervicitis in FSW.

The objectives of this observational, cross-sectional study were to describe the prevalence and etiologies of cervicitis in FSW receiving care at a public health clinic and associated mobile clinic in Callao-Lima, Peru. We also sought to identify sociodemographic, behavioral and biological factors associated with cervicitis. In particular, we sought to determine if an association between BV and cervicitis existed in these women, particularly given the high reported rates of BV in Peruvian FSW [11].

## Methods

### Setting, study participants & enrolment

In Peru, sex work is permitted by FSW who are registered at a public health clinic and are of 18 years of age or older. Registration requires monthly health assessments which are free of charge, including treatment [11]. Approximately two-thirds of Lima’s estimated 15,000 FSW are registered. The Centro de Salud ‘Alberto Barton’ (CSAB), located in the port city of Callao-Lima, provides healthcare to registered FSW, while an associated mobile clinic provides care to unregistered FSW. These monthly healthcare checks are provided by Peruvian Ministry of Health clinicians, and include genital examination and collection of cervical and vaginal swabs for basic onsite microbiology (including Gram stain, wet mount and KOH staining) in addition to gonococcal culture. Syphilis screening (by non-treponemal assay) is performed quarterly and HIV testing is performed twice per year. If required, much of the treatment is syndromic as per the National STI Guidelines [11].

FSW presenting for medical assessment at either of these clinics between November 2008 and January 2011 were invited to participate in this cross-sectional study. Unregistered and registered FSW were eligible to participate, with registration status determined on routine assessment. Unregistered FSW were offered enrolment (if eligible) when presenting for routine assessment at the mobile clinic. FSW 18 years of age or older were eligible for inclusion. Informed consent was obtained from all study participants. The study was approved by the Institutional Review Boards of the University of Washington, Universidad Nacional Mayor de San Marcos, the Directorate of Callao, and US Naval Medical Research Unit No. 6 (NAMRU-6), Callao, Peru.

### Study procedures

All participants completed a face-to-face questionnaire to provide data on sociodemographic and behavioral variables and underwent genital examination with collection of vaginal, endocervical and blood samples. Genital examination was performed by one of the regular clinic doctors as an assigned study physician. Cervicitis was defined as the presence of mucopus and friability in concurrence with the Centers for Disease Control and Prevention recommendations [3]. PID was defined as cervicitis in the presence of lower abdominal pain plus cervical motion tenderness, adnexal tenderness or uterine tenderness.

Saline wet-mount microscopy was performed on cervical samples for detection of *Trichomonas vaginalis* (TV) at an on-site laboratory at CSAB. Further cervical specimen testing occurred at NAMRU-6 laboratories in Lima, Peru. One cervical sample was placed in specimen transport medium (Digene Diagnostics, Silver Spring, MD) for molecular testing of *Chlamydia trachomatis* (CT), *Neisseria gonorrhoea* (GC) and Human Papillomavirus (HPV). CT and GC testing was performed using Amplicor CT/GC PCR according to the manufacturer’s instructions (Roche, Pleasanton, California, USA). HPV DNA was extracted and PCR analysis performed according to methods described elsewhere [14]. Vaginal swabs were evaluated with Gram stain, KOH and saline wet mount tests for the detection of candidiasis, TV and BV. In accordance with recently standardised research definitions, BV was diagnosed by positive Amsel criteria and Nugent scoring [15]. All vaginal swab testing occurred at CSAB.

Blood samples were transported to NAMRU-6 laboratories for analysis. Serum was screened for HIV using a Vironostika HIV Ag/Ab assay (bioMeriéux, Marcy l′Etoile, France); positive assays were confirmed by line immunoblot assay (INNO-LIA HIV I/II Score, Innogenetics, Gent, Belgium). Serum samples were also tested for HTLV-1/2 antibody by ELISA assay (BioElisa HTLV I/II 5.0 BioKit, Llica d’Amunt, Barcelona, Spain) with confirmatory testing using line immunoblot assay (HTLV I/II score, Innogenetics, Gent, Belgium). Serum was screened for syphilis using Rapid Plasma Reagin (RPR-nosticon II, bioMeriéux, Marcy l′Etoile, France) and confirmed by Treponema Pallidum Hemagglutination Assay (TPHA; Syphagen TPHA - Biokit, Llica d’Amunt, Barcelona, Spain). Women with positive STI test results were counselled and treated according to Peruvian Ministry of Health Guidelines [11].

### Statistical analysis

All data were double entered into an electronic database and analyzed using STATA, version 12.0 (StataCorp, College Station, TX). Bivariate associations of cervicitis with sociodemographic, behavioral, and biological variables were examined using Chi-squared tests to determine significant differences between groups. Logistic regression was used to determine whether sociodemographic, behavioral, or other sexual health-related characteristics were associated with the diagnosis of cervicitis. Both unadjusted and adjusted logistic models were fit to estimate odds ratios (OR) and 95% confidence intervals (95% CI). For sociodemographic variables, we used the criterion of a greater than 10% change in the OR to identify confounders in adjusted models. For biological variables, we specified the presence of genital tract infections associated with both cervicitis and bacterial vaginosis (namely CT, GC or TV) as *a priori* confounders in an adjusted model. A significance level of 0.05 was used for all hypothesis tests.

## Results

A total of 467 FSW were enrolled. The presence of cervicitis was unable to be assessed in 70 women due to menses, pessary use, hysterectomy or other examination difficulties. Cervicitis was present in 24.9% (99/397) of the remaining participants. Table 1 presents the sociodemographic, behavioral and biological characteristics, including frequency of STI and other genital tract infections, in those FSW with and without cervicitis. In the women with cervicitis, CT was present in 4.6% (4/87), TV in 4.0% (4/99), GC in 0% (0/87) and no pathogen was detected on cervical microbiology in 91.9% (91/99). There were nil cervical co-infections detected. Bacterial vaginosis was detected on vaginal microbiology in 36.9% (31/84) of cervicitis cases for whom a definitive BV diagnosis could be made. Bivariate analyses of associations between sociodemographic and biological variables with cervicitis are presented in Table 2. Regular use of healthcare services and Ecuadorian nationality were significantly associated with a reduced risk of cervicitis, even after multivariate analysis. BV was more common in women with cervicitis, however this association did not reach statistical significance (aOR = 1.47 [0.87, 2.48], p = 0.15).

**Table 1:** **Sociodemographic, behavioral and biological characteristics in FSW with and without cervicitis**^**a**^

	**FSW with cervicitis (*n*** **= 99)**		**FSW without cervicitis (*n*** **= 298)**		***p*-value**
Age, median years (IQR^b^)	30 (24–38)		27 (23–33)		0.02
Age at initiation of sex work, median years (IQR)	22 (20–27)		22 (19–27)		0.32
Time in sex work, median months (IQR)	36 (10–132)		24 (6–72)		0.03
	*n*	%	*n*	%	
Nationality					0.01
Peruvian	90	90.9	228	76.5	
Other countries^c^	9	9.1	70	23.5	
Registration status					0.01
Registered	40	40.4	102	34.2	
Unregistered	33	33.3	55	18.5	
Place of work					0.07
Legal brothel	40	65.6	178	77.1	
Other^d^	21	34.4	53	22.9	
Number of clients in last week					0.04
None	35	36.1	101	35.7	Ref
1 to 10	29	29.9	59	20.9	0.24
11 to 20	19	19.6	35	12.4	0.19
21 to 40	7	7.2	43	15.2	0.10
> 40	7	7.2	45	15.9	0.08
Most frequent sexual practices with clients					0.42
Vaginal	8	8.3	12	4.2	0.14
Vaginal and oral	70	72.9	211	74.6	Ref
Vaginal, oral and anal	18	18.8	59	20.9	0.78
Has a non-client sexual partner	65	34.3	170	57.8	0.17
Oral contraceptive pill use	19	80.8	52	17.7	0.74
Condom use with clients					0.21
Always	75	78.0	234	83.0	Ref
Between rarely and almost always	10	10.5	15	5.3	0.09
Never	10	10.5	33	11.7	0.88
Sexually transmitted/genital tract infections					
Syphilis	0	0.0	5	1.7	0.20
*Chlamydia trachomatis* (CT)	4	4.6	11	4.9	0.91
*Neisseria gonorrhea* (GC)	0	0.0	1	0.4	0.53
*Trichomonas vaginalis* (TV)	4	4.0	2	0.7	0.02
Human Papillomavirus (HPV)	48	48.5	140	47.0	0.80
Human Immunodeficiency Virus (HIV)	1	1.0	2	0.7	0.74
Human T-cell Lymphotropic Virus (HTLV)	3	3.0	5	1.7	0.41
Bacterial vaginosis	31	36.9	74	28.0	0.12
Candidiasis	7	7.1	19	6.5	0.83
Clinical diagnoses and syndromes					
Vaginal discharge	48	50.0	151	59.5	0.11
Genital ulcer	0	0.0	3	1.0	0.32
Pelvic Inflammatory Disease (PID)	4	4.0	9	3.0	0.62

^a^Numbers may not sum to total *n* due to missing values.

^b^Inter-quartile range.

^c^Ecuador (n = 63), Colombia (n = 11), Dominican Republic (n = 3).

^d^Included clandestine brothels, bars, hostels, street and telephone.

**Table 2:** Associations between cervicitis and sociodemographic, behavioral and biological characteristics

**Characteristic**	**Unadjusted OR (95%CI)**	***p*value**	**Adjusted OR (95%CI)**	***p*value**
Age (years)	1.03 (1.00, 1.06)	0.02	1.01 (0.99, 1.04)^a^	0.32
Age at first sex	1.03 (0.94, 1.13)	0.53	1.02 (0.93, 1.12)^a^	0.66
Age of starting sex work	1.02 (0.98, 1.05)	0.33	0.99 (0.96, 1.03)^a^	0.72
Drug and/or alcohol use	1.01 (0.56, 1.82)	0.98	0.79 (0.43, 1.44)^a^	0.44
Earnings per client	1.00 (1.00, 1.01)	0.11	1.00 (1.00, 1.01)^a^	0.13
History of STI	1.26 (0.75, 2.09)	0.38	1.20 (0.70, 2.05)^a^	0.52
Illegal workplace	1.76 (0.96, 3.25)	0.07	1.48 (0.77, 2.84)^a^	0.24
Registered	0.65 (0.37, 1.15)	0.14	1.58 (0.63, 4.01)^g^	0.33
Months doing sex work	1.00 (1.00, 1.01)	0.03	1.00 (1.00, 1.01)^a^	0.1
Regularly receives medical care	0.48 (0.30, 0.77)	<0.01	0.54 (0.34, 0.87)^a^	0.01
Has a non-client sexual partner	1.39 (0.87, 2.24)	0.17	1.42 (0.86, 2.33)^a^	0.17
Number of clients per week				
None	1.00 (Ref)	_	1.00 (Ref)	_
1 to 10	1.42 (0.79, 2.55)	0.24	1.33 (0.73, 2.41)^b^	0.35
11 to 20	1.57 (0.80, 3.09)	0.19	1.45 (0.72, 2.90)^b^	0.3
21 to 40	0.47 (0.19, 1.14)	0.1	0.42 (0.17, 1.04)^b^	0.06
> 40	0.45 (0.19, 1.09)	0.08	0.49 (0.20, 1.20)^b^	0.12
Condom use with clients (always)	0.77 (0.43, 1.38)	0.38	0.84 (0.46, 1.54)^a^	0.58
Condom use with clients or non-client partners (never)	1.35 (0.42, 4.33)	0.62	_	_
Condom use with non-client partners (always)	0.95 (0.82, 1.09)	0.48	0.50 (0.17, 1.48)^c^	0.21
Use of oral contraceptive pill	1.11 (0.62 - 1.98)	0.74	1.33 (0.72, 2.45)^a^	0.36
Education				
Primary or less	0.95 (0.41, 2.17)	0.9	0.87 (0.36, 2.10)^a^	0.76
Secondary, incomplete	1.02 (0.61, 1.70)	0.94	0.98 (0.55, 1.72)^a^	0.93
Secondary complete	1.00 (Ref)	_	1.00 (Ref)	_
Technical School	0.74 (0.29, 1.64)	0.41	0.62 (0.25, 1.53)^a^	0.3
University	0.63 (0.23, 1.70)	0.36	0.47 (0.15, 1.44)^a^	0.19
Nationality				
Peruvian	1.00 (Ref)	_	1.00 (Ref)	_
Ecuadorian	0.27 (0.11, 0.64)	<0.01	0.31 (0.13, 0.76)^a^	0.01
Columbian or Dominican	0.8 (0.22, 2.94)	0.74	0.67 (0.18, 2.47)^a^	0.54
Sexual practices				
Vaginal/Oral	1.00 (Ref)		1.00 (Ref)	
Vaginal	2.05 (0.81, 5.17)	0.13	1.95 (0.72, 5.30)^a^	0.19
Vaginal/Oral/Anal	0.87 (0.48, 1.57)	0.65	0.84 (0.45, 1.56)^a^	0.58
Genital tract infections				
Bacterial Vaginosis (BV)	1.50 (0.89, 2.52)	0.12	1.47 (0.87, 2.48)^f^	0.15
*Chlamydia trachomatis* (CT)	0.94 (0.29, 3.03)	0.91	_	_
Any cervicitis-causing STI^d^	1.78 (0.72, 4.39)	0.21	_	_
Human Papillomavirus	0.95 (0.59, 1.53)	0.84	0.96 (0.60, 1.55)^f^	0.87
HTLV^e^	1.83 (0.43, 7.81)	0.41	1.90 (0.45, 8.12)^f^	0.39
Pelvic Inflammatory Disease	1.35 (0.41, 4.49)	0.62	1.34 (0.40, 4.45)^f^	0.64
Vaginal discharge	0.68 (0.43, 1.09)	0.11	0.67 (0.42, 1.08)^f^	0.10

^a^Adjusted for receipt of regular medical care and nationality.

^b^Adjusted for nationality.

^c^Adjusted for age, earnings, and regular medical care.

^d^*C. Trachomatis, N. Gonorrhoea, T. vaginalis.*

^e^Human T-cell Lymphotropic Virus.

^f^Adjusted for presence of any cervicitis-causing STI.

^g^Adjusted for receipt of regular medical care.

Further analyses were conducted to examine the association between cervicitis and regular clinic attendance, Ecuadorian nationality, and BV. The most common STI associated with cervicitis, namely CT, GC and TV, were less common in regular clinic attendees (OR = 0.39 [0.15, 0.99], p = 0.04). BV was also less common in regular clinic attendees (OR = 0.39, 95% [0.24, 0.62] p < 0.001). Regular clinic attendance was more common in Ecuadorian FSW (OR = 2.72, [1.49, 4.94], p = 0.001).

## Discussion

Almost one quarter of FSW in this study had cervicitis, second only to vaginal discharge as the most common syndrome. Cervicitis in this study was predominantly non-gonococcal, non-chlamydial in etiology, and no pathogen was detected in cervical samples in over 90% of participants with cervicitis. Cervicitis that is not associated with chlamydial or gonoccocal infections is the most common type of cervicitis overall and is referred to as ‘non-specific cervicitis’ (NSC) [1]. The rate of NSC determined here was higher than some rates reported in non-FSW women [6].

Studies in other FSW populations have demonstrated a strong association between gonoccoal, chlamydial or *Trichomonas* infection with clinical manifestations of cervicitis [8,9]. No such association was found in the women studied here, and this likely reflects the low overall prevalence of STI diagnosed, in contrast to the higher STI prevalence reported in FSW in other Latin American countries, Asia and Africa [8,9,11,16,17].

Other potential reasons for the high frequency of NSC and lack of association with typical cervicitis-causing organisms may include ongoing inflammation of the cervix after previously treated chlamydial or gonorrhoeal infections, perhaps mediated by an abnormal immune response [2]. Data was unfortunately not collected on prior antimicrobial treatment for cervicitis or other genital tract syndromes in these women, which is a significant limitation to this study.

Cervical irritation due to douches and spermicides may also have contributed to high NSC rates [2]. While no data was collected on the use of spermicides or frequency of douching, douching is common in this population (personal observation by authors). Additionally, testing for other potential pathogens associated with cervicitis such as HSV-2 and *M. genitalium* were not performed in this study [1]. Furthermore, microscopy was used for the detection of trichomoniasis in this study, a method that is less sensitive compared to culture-based, molecular and immunochromatographic methods [3]. This relatively insensitive method of TV detection is a limitation of this study, and future studies should consider the use of newer generation diagnostic methods such as PCR.

Bacterial vaginosis may play a causative role in some NSC cases. BV was the second most common genital tract infection in our study and was present in over a third (36.9%) of cervicitis cases. On bivariate analysis, the association between BV and cervicitis failed to reach significance, but this may reflect insufficient power due to the number of FSW enrolled. There is growing evidence from other studies that BV is an independent risk factor for cervicitis [6]. Bacterial vaginosis could lead to cervicitis through a loss of bactericidal H_2_0_2_-producing lactobacilli, reduced levels of protective vaginal mucins, and increased pro-inflammatory enzymes and cytokines, which in turn may decrease the cervical mucus barrier [6].

Regular clinic attendance was associated with a 46% reduced risk of cervicitis, possibly due to the reduced rates of STI and BV noted in the regular clinic attendees. This may reflect frequent and early antibiotic treatment of genital tract syndromes and infections, although the exact frequency of antibiotic treatment was not determined. Self-treatment with antimicrobials was also considered as a confounding factor in women who attended clinics, however only one participant reported receiving treatment outside of clinics (data not shown). Preventative STI education delivered during clinic attendance may also contribute to the protective effect associated with regular clinic attendance. Regardless of the mechanism, the current Peruvian Ministry of Health program of free monthly health checks for FSW may be effective for reducing rates of cervicitis, BV and cervicitis-causing STI noted in FSW with regular clinic attendance.

Ecuadorian nationality was also protective against cervicitis, even after controlling for regular clinic attendance, which was more common in the Ecuadorian participants. The effect of Ecuadorian nationality may reflect unmeasured cultural differences in sexual practices, douching or other risk behaviors for cervicitis and STI.

## Conclusion

In conclusion, cervicitis was a common condition in Peruvian FSW and in the context of very low STI rates was predominately non-specific cervicitis. Our findings may not be generalizable to other populations of FSW, particularly those with higher rates of STI, but encourage further evaluation of the etiologies and treatment of cervicitis in FSW populations. The protective effect of Ecuadorian nationality is intriguing, and future studies could examine the effect of migration upon the sexual health and risk behaviors of FSW. Defining the role of *M. genitalium*, HSV and other emerging pathogens such as *Ureaplasma urealyticum,* adenovirus and cytomegalovirus in FSW with cervicitis may also reveal new associations [2,3].

## Competing interests

The authors declare that they have no competing interests.

## Authors’ contributions

SP developed manuscript and assisted in data analysis. MC performed data collection and assisted in study logistics, data analysis and manuscript development. KH analyzed data and contributed to manuscript development. VS assisted in study logistics, data collection and manuscript development. SM and JZ developed study design and logistics, assisted in data analysis and assisted in manuscript development. All authors read and approved the final manuscript.

## Pre-publication history

The pre-publication history for this paper can be accessed here:

http://www.biomedcentral.com/1471-2334/13/195/prepub

## Disclaimer

The views expressed in this article are those of the authors and do not necessarily reflect the official policy or position of the Department of the Navy, Department of Defense, nor the U.S. Government. Dr SM Montano is a US Government Employee. This work was prepared as part of her official duties. Title 17 U.S.C. §105 provides that ‘Copyright protection under this title is not available for any work of the United States Government.’ Title 17 U.S.C. §101 defines a U.S. Government work as a work prepared by a military service member or employee of the U.S. Government as part of that person’s official duties.

## References

[B1] Lusk MJ Konecny P Cervicitis: a review Curr Opin Infect Dis 2008 21 1 49 55 18192786 10.1097/QCO.0b013e3282f3d988

[B2] Marrazzo JM Martin DH Management of women with cervicitis Clin Infect Dis 2007 44 Suppl 3 S102 S110 17342663 10.1086/511423

[B3] Workowski KA Berman S Sexually transmitted diseases treatment guidelines MMWR Recomm Rep 2010 59 RR-12 1 110 21160459

[B4] Zunt JR Cervical shedding of human T cell lymphotropic virus type I is associated with cervicitis J Infect Dis 2002 186 11 1669 1672 10.1086/345364 12447745 PMC2675941

[B5] Kreiss J Association between cervical inflammation and cervical shedding of human immunodeficiency virus DNA J Infect Dis 1994 170 6 1597 1601 10.1093/infdis/170.6.1597 7996003

[B6] Marrazzo JM Risk factors for cervicitis among women with bacterial vaginosis J Infect Dis 2006 193 5 617 624 10.1086/500149 16453256

[B7] Ryan KA Prevalence and prediction of sexually transmitted diseases among sex workers in Cameroon Int J STD AIDS 1998 9 7 403 407 10.1258/0956462981922494 9696196

[B8] Pepin J Mycoplasma genitalium: an organism commonly associated with cervicitis among west African sex workers Sex Transm Infect 2005 81 1 67 72 10.1136/sti.2003.009100 15681727 PMC1763741

[B9] Mukenge-Tshibaka L Syndromic versus laboratory-based diagnosis of cervical infections among female sex workers in Benin: implications of nonattendance for return visits Sex Transm Dis 2002 29 6 324 330 10.1097/00007435-200206000-00003 12035021

[B10] Behets FM Evidence-based treatment guidelines for sexually transmitted infections developed with and for female sex workers Trop Med Int Health 2003 8 3 251 258 10.1046/j.1365-3156.2003.01017.x 12631316

[B11] Estrategia Sanitaria Nacional Prevención y Control de Infecciones de Transmisión Sexual y VIH-SIDA-Lima Guía Nacional de Manejo de Infecciones de Transmisión Sexual 2006 Peru: Ministerio de Salud

[B12] Morton AN An outreach programme for sexually transmitted infection screening in street sex workers using self-administered samples Int J STD AIDS 1999 10 11 741 10.1258/0956462991913286 10563563

[B13] Dallabetta GA Problems, solutions, and challenges in syndromic management of sexually transmitted diseases Sex Transm Infect 1998 74 S1 10023346

[B14] Gravitt PE Genotyping of 27 human papillomavirus types by using L1 consensus PCR products by a single-hybridization, reverse line blot detection method J Clin Microbiol 1998 36 10 3020 3027 9738060 10.1128/jcm.36.10.3020-3027.1998 PMC105104

[B15] Marrazzo JM Bacterial vaginosis: identifying research gaps. Proceedings of a workshop sponsored by DHHS/NIH/NIAID November 19–20, 2008 Sex Transm Dis 2010 37 12 732 744 10.1097/OLQ.0b013e3181fbbc95 21068695 PMC3137891

[B16] Nguyen TV Sexually transmitted infections and risk factors for gonorrhea and chlamydia in female sex workers in Soc Trang, Vietnam Sex Transm Dis 2008 35 11 935 940 10.1097/OLQ.0b013e3181812d03 18685547 PMC2903543

[B17] Bautista CT Sexual practices, drug use behaviors, and prevalence of HIV, syphilis, hepatitis B and C, and HTLV-1/2 in immigrant and non-immigrant female sex workers in Argentina J Immigr Minor Health 2009 11 2 99 104 10.1007/s10903-007-9114-2 18175218

